# The Determination of the Biocompatibility of New Compositional Materials, including Carbamide-Containing Heterocycles of Anti-Adhesion Agents for Abdominal Surgery

**DOI:** 10.3390/molecules29040851

**Published:** 2024-02-14

**Authors:** Nurdana Kanasheva, Dmitry A. Fedorishin, Maria V. Lyapunova, Mikhail V. Bukterov, Olga A. Kaidash, Abdigali A. Bakibaev, Rakhmetulla Yerkassov, Togzhan Mashan, Rimma Nesmeyanova, Vladimir V. Ivanov, Elena V. Udut, Vera P. Tuguldurova, Margarita V. Salina, Victor S. Malkov, Alexey S. Knyazev

**Affiliations:** 1 Department of Chemistry, L.N. Gumilyov Eurasian National University, Astana 010008, Kazakhstan; erkass@mail.ru (R.Y.); togzhan-mashan@mail.ru (T.M.); 2 Faculty of Chemistry, National Research Tomsk State University, Tomsk 634028, Russia; strix187@yandex.ru (D.A.F.); lyapunova.mari@mail.ru (M.V.L.); bakibaev@mail.ru (A.A.B.); tuguldurova91@mail.ru (V.P.T.); 89991237185@mail.ru (M.V.S.); malkov.tsu@yandex.ru (V.S.M.); kas854@mail.ru (A.S.K.); 3 Central Research Laboratory, Siberian State Medical University, Tomsk 634050, Russia; bukterov.mv@ssmu.ru (M.V.B.); kaidash_2011@mail.ru (O.A.K.); ivanovvv1953@gmail.com (V.V.I.); evu8@mail.ru (E.V.U.); 4 Department of Chemistry and Chemical Technologies, Faculty of Natural Sciences, Toraighyrov University, Pavlodar 140008, Kazakhstan; nesm_r@mail.ru

**Keywords:** anti-adhesion agents, carbamide-containing heterocycles, composite materials, barrier films, biocompatibility, surgery

## Abstract

Due to traumatic injuries, including those from surgical procedures, adhesions occur in over 50% of cases, necessitating exclusive surgical intervention for treatment. However, preventive measures can be implemented during abdominal organ surgeries. These measures involve creating a barrier around internal organs to forestall adhesion formation in the postoperative phase. Yet, the effectiveness of the artificial barrier relies on considerations of its biocompatibility and the avoidance of adverse effects on the body. This study explores the biocompatibility aspects, encompassing hemocompatibility, cytotoxicity, and antibacterial and antioxidant activities, as well as the adhesion of blood serum proteins and macrophages to the surface of new composite film materials. The materials, derived from the sodium salt of carboxymethylcellulose modified by glycoluril and allantoin, were investigated. The research reveals that film materials with a heterocyclic fragment exhibit biocompatibility comparable to commercially used samples in surgery. Notably, film samples developed with glycoluril outperform the effects of commercial samples in certain aspects.

## 1. Introduction

Due to traumatic injuries to internal organs and an increasing number of surgical procedures, the prevalence of abdominal adhesions is on the rise. Adhesions’ development is linked to chronic abdominal pain syndrome, which significantly diminishes patients’ quality of life, impairs reproductive function in women, and can lead to acute adhesive intestinal obstruction. Currently, the exclusive method for treating adhesions involves surgical intervention through mechanical dissection. However, preventive measures can be implemented during abdominal organ surgeries. Over the past decades, a crucial role in preventing abdominal adhesions has been played by a category of specialized products known as anti-adhesion barriers [1,2]. These barriers are constructed from reactive, biodegradable materials. An ideal barrier, characterized by high safety and efficacy, should not induce inflammation or immune responses, persist throughout the critical remesothelization phase, remain securely in place without the need for stitches or staples, stay active in the presence of blood, and be fully resorbable. Additionally, it should not impede the healing process, trigger infections, contribute to oncologic processes, or promote the formation of new adhesions. To achieve optimal results, these barrier agents should be administered intraperitoneally either during or at the conclusion of surgery [3].

Barrier agents function primarily by targeting inflammation reduction and exudation in wound areas, temporarily delineating wound surfaces, preventing fibrin accumulation in the abdominal cavity, inhibiting fibroblast proliferation, and providing fermentative protection to tissues against hypoxia damage [4]. The utilization of anti-adhesion barriers has been demonstrated as an etiopathogenetic approach to prevent adhesion formation. These barriers safeguard the wound from mechanical damage and adhesion with neighboring organs while also promoting favorable effects on reparative tissue regeneration [5]. Presently, a variety of “barrier type” anti-adhesion agents are available, including those based on icodextrin, polytetrafluoroethylene, hyaluronic acid, polyethylene oxide, and cellulose.

Dextran-70 underwent testing as an anti-inflammatory agent, yielding positive experimental and clinical outcomes in the 1980s. Despite these promising results, practical application revealed reported side effects, including interstitial edema, ascites, and coagulopathy. Consequently, the approval for the use of dextran as an anti-inflammatory agent was not granted, leading to its current limited usage [6].

Constructed from polytetrafluoroethylene, a material commonly utilized in vascular grafts known for its low thrombosis factor, the anti-adhesion “barrier” Preclude (Gore-Tex Surgical Membrane, W. L. Gore & Associates, Flagstaff, AZ, USA) was developed. While this product received positive evaluations in a study and was endorsed for gynecologic surgeries by The Myomectomy Adhesion Study Group in 1995, it does have certain drawbacks that restrict its application. Primarily, these drawbacks are associated with its hydrophobic nature, leading to inadequate tissue adhesion. Additionally, the material lacks biodegradability. Consequently, this “barrier” necessitates fixation with threads and persists as a foreign body in the abdominal cavity indefinitely, heightening the risk of adhesion and infection development in the extended postoperative period [7].

In clinical studies, both hyaluronidase alone and its compounds with iron and carboxymethylcellulose (CMC) have demonstrated efficacy. Barriers incorporating hyaluronidase, besides serving as anti-adhesion agents, also exhibit anti-inflammatory effects and enhance the proliferation of mesothelial cells [8,9]. Among hyaluronidase-based agents, the compound with CMC and two anionic polysaccharides, known as Seprafilm (Genzyme Corporation, Cambridge, MA, USA), is more commonly utilized. This bioabsorbable membrane is non-toxic, non-immunogenic, and biocompatible, functioning as a film that covers traumatized surfaces. Although the membrane transforms into a gel within 24–48 h, it remains at the placement site for up to 7 days and completely resorbs by day 28, eliminating the need for stitches. It proves effective even in the presence of blood. Seprafilm has substantially reduced the extent and severity of postoperative adhesions in various experiments and randomized clinical trials in gynecology and general surgery. However, its potential to reduce the incidence of adhesive intestinal obstruction remains uncertain [10]. Additionally, its relatively high cost and the modified hyaluronic acid in the drug may pose risks, potentially leading to anastomosis failure and the formation of intra-abdominal abscesses [11].

Oxidized regenerated cellulose, recognized as the Interceed antiadhesion barrier (Ethicon Inc., Somerville, NJ, USA), is a membrane that fully absorbs within a span of 28 days. This drug has been employed in gynecologic surgeries since the late 1980s, and both experimental and clinical applications have indicated a reduction in the frequency and severity of postoperative adhesions. However, its widespread utilization is constrained by diminished efficacy in the presence of blood or excess peritoneal fluid. The necessity for achieving comprehensive hemostasis before employing Interceed is attributed to the deposition of fibrin between tissue fibers [12].

Hence, the independent application of local and general anti-inflammatory agents in the majority of clinical trials has not yielded satisfactory outcomes, and certain drugs have not progressed beyond the experimental study phase [13].

**Figure 1:**
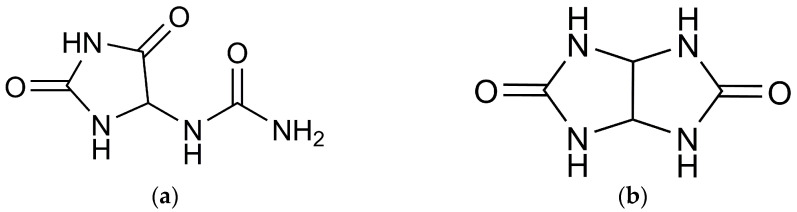
Chemical structures of allantoin (**a**) and glycoluril (**b**).

When seeking the “ideal” anti-adhesion agent, it is crucial to consider the biological parameters of the utilized reagents. In our study, during the selection of the composition for the barrier membrane, we investigated compounds that include the urea-containing heterocyclic fragment of allantoin and glycoluril (Figure 1).

Allantoin (Figure 1a) belongs to the well-established class of azaheterocycles in the imidazolidinone series, specifically hydantoins. The beneficial biological and pharmacological properties of this compound have been extensively discussed in several review articles [14,15,16]. Allantoin, chemically defined as (2,5-Dioxo-4-imidazolidinyl) urea, is a heterocyclic compound with a five-membered cycle containing a carbamide substituent in the 4th position. Presently, allantoin is a component in over 1300 different cosmetic products [14]. Recognized for its regenerative effects, it aids in the removal of abrasions and scars [17]. When included in creams, it safeguards the skin from sunburn, weathering, and cracking, offering gerontological benefits [18] and restoring normal moisture and elasticity to the skin. Allantoin has the capability to diminish the genotoxic effects of ultraviolet radiation [19]. Additionally, research has demonstrated its ability to inhibit various destructive processes induced by reactive oxygen species, thereby exhibiting antioxidant properties.

Glycoluril (Figure 1b), specifically (2,4,6,8-tetraazabicyclo[3.3.0]octane-3,7-dione), serves as the precursor to a class of nitrogen-containing heterocycles known as bicyclic bisureas of the octane series. The distinctive structural framework of these bicyclic bisureas, or glycoluriles, has made them the foundation for the development of valuable substances utilized in various human activities, including disinfectants, drugs [20,21], and polymer stabilizers [21]. Notably, glycoluriles have been identified as key components in the construction of polycyclic condensed systems, such as cucurbit[n]urils [22] and bambus[n]urils [23], each exhibiting unique physicochemical properties.

The commonality between allantoin and glycoluril lies in their nature as carbamide-containing heterocycles. Owing to their polyfunctionality and intricate chemical structures, both compounds showcase a broad spectrum of physiological activities and a propensity for polycondensation processes.

Film materials were formulated using these compounds and subsequently studied for cytotoxicity, adhesion to blood proteins, adhesion to macrophages, antibacterial activity, antioxidant activity, and hemocompatibility.

## 2. Results

Throughout this investigation, novel composite materials derived from Na-CMC and carbamide-containing heterocycles (allantoin and glycoluril) were synthesized, and their biocompatibility was assessed using the test models described below. The synthesis of allantoin and glycoluril followed previously established methods [24,25], and the procedure for preparing the composite material is detailed in the Section 4 of this study.

### 2.1. Evaluation of Hemocompatibility of Composite Film Materials

Through experiments, it was determined that in all samples, when incubating erythrocyte suspensions from five patients in three measurements with extracts of Seprafilm™ (Genzym Corporation, Cambridge, MA, USA) as the comparison sample (**1**) and the investigated composite film materials based on allantoin (**2**, **3**) and glycoluril (**4**, **5**), the hemolysis value did not exceed 2% (Table 1).

Therefore, the experimental film samples exhibit no cytotoxic effects on erythrocytes, and their hemolytic activity is comparable to that of the reference sample Seprafilm™ (**1**).

Considering the aforementioned information, to identify the factors contributing to the low level of hemolysis and, consequently, the high hemocompatibility of the examined samples, the adsorption of blood plasma proteins on the surface of the studied composite film materials was investigated.

### 2.2. Adsorption of Blood Plasma Proteins on the Surface of the Investigated Composite Film Materials

Following the experiments, it was determined that the protein concentrations in donor serum diluted ten times were 7.11 g/L, 6.85 g/L, 5.85 g/L, 6.14 g/L, and 6.83 g/L, respectively. Subsequent to a 2 h incubation of serum at 37 °C in wells containing the films under examination, there was a marginal decrease in protein concentration attributed to protein adsorption.

According to the information presented in Table 2, protein adsorption in film (**1**) was 2.96 ± 0.13%. The protein adsorption for the investigated composite film materials, based on glycoluril- and allantoin-modified sodium salt of carboxymethylcellulose (**2**–**5**), was 3.06 ± 0.17% (*p* = 0.917), 3.29 ± 0.11% (*p* = 0.117), 3.16 ± 0.11% (*p* = 0.347), and 3.28 ± 0.17% (*p* = 0.117), respectively. These values were not significantly different from the protein adsorption capacity of sample (**1**).

As indicated by the information in Table 2, protein adhesion observed in the samples of the investigated composite film materials, based on the sodium salt of carboxymethylcellulose modified with glycoluril and allantoin (**2**–**5**), is comparable to protein adhesion in the reference sample “Seprafilm” (**1**).

### 2.3. Evaluation of Cytotoxicity

**Figure 2:**
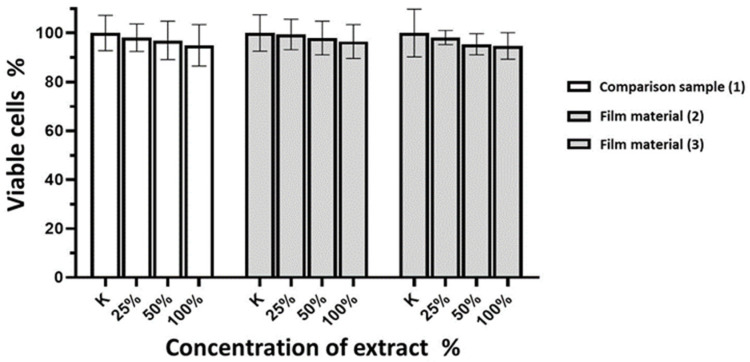
Cell viability of 3T3-L1 normal fibroblast culture (in %) after incubation with different concentrations of extracts of commercial sample of Seprafilm™ membrane and investigated films.

The experiments revealed that incubating the 3T3-L1 normal fibroblast cell line with various concentrations of extracts from the investigated samples of composite film materials, based on allantoin-modified sodium salt of carboxymethylcellulose (**2**, **3**), for 24 h, as well as the reference sample (**1**), did not result in a noteworthy reduction in cell viability in the neutral red test (Figure 2).

**Figure 3:**
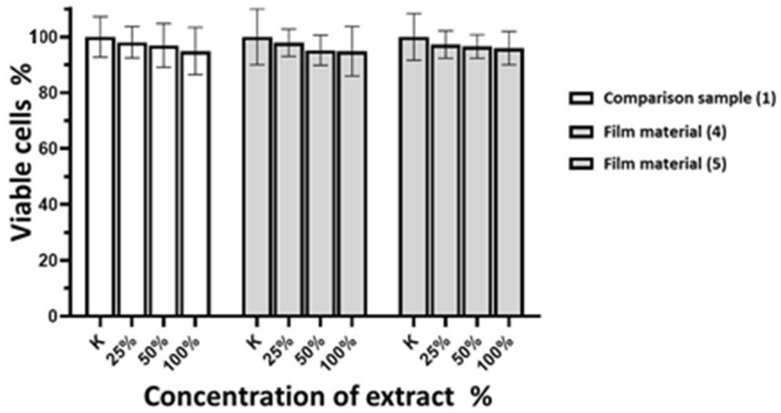
Cell viability of 3T3-L1 normal fibroblast culture (in %) after incubation with different extract concentrations of Seprafilm™ commercial membrane sample and the tested films.

Culturing the 3T3-L1 normal fibroblast cell line with varying extract concentrations from the examined samples of composite film materials, which are based on glycoluril-modified sodium salt of carboxymethylcellulose (**4**, **5**), for 24 h similarly showed no impact on cell viability (Figure 3).

Therefore, extracts from the examined films, as well as extracts from the reference film, do not exhibit a notable cytotoxic effect on the 3T3-L1 fibroblast cell culture.

**Figure 4:**
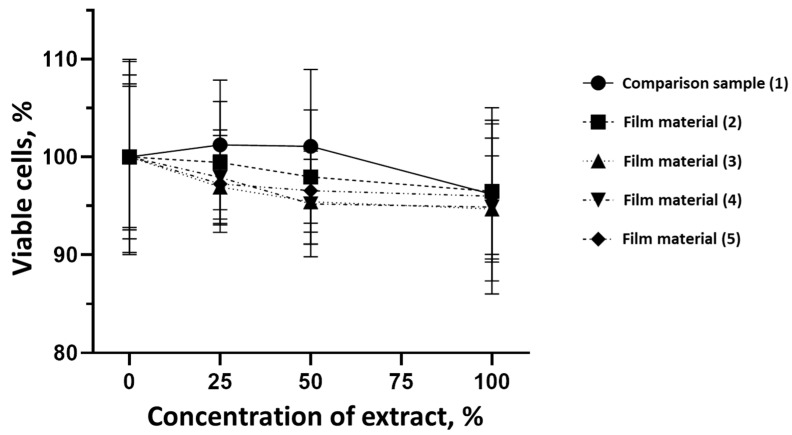
Impact of different extract concentrations of commercial Seprafilm™ membrane sample and tested film samples.

A comparative examination of the cytotoxicity of the studied composite film samples (**2**–**5**) and the reference sample (**1**) is detailed in Table 3 and Figure 4. The findings suggest that the cytotoxicity of the investigated composite film materials, based on the sodium salt of carboxymethylcellulose modified with glycoluril and allantoin (**2**–**5**), is comparable to the cytotoxicity of the commercial sample (**1**).

### 2.4. Evaluation of Macrophage Adhesion to the Surface of the Tested Samples

Following the investigation of cytotoxicity, the adhesion of macrophages to the surface of the examined samples was explored. In the experiments, it was observed that the number of adhered macrophages from the RAW 264.7 line on the surface of tablet wells without the investigated composite film materials in the microscope’s field of view was 355 ± 36 (Table 4). Upon placing RAW 264.7 cells in the tablet wells with the surface of the comparison sample “Seprafilm”, the number of adhered cells significantly decreased to 128 ± 16 within the microscope’s view field. The adhesion of macrophages on the surface of the composite film materials samples, based on the sodium salt of carboxymethylcellulose modified by allantoin (**2**, **3**), did not differ significantly from the comparison sample “Seprafilm”.

The number of adhered cells in the microscope’s field of view was 215 ± 31 (*p* = 0.058) and 195 ± 29 (*p* = 0.064) cells, respectively. In contrast, the ability of macrophages to adhere in the samples of composite film materials based on glycoluril-modified sodium salt of carboxymethylcellulose (**4**, **5**) was significantly lower than in sample (**1**) and the studied samples (**2**, **3**). The number of adhered cells in the microscope’s field of view was 61 ± 8 and 18 ± 5, respectively.

Consequently, the tested composite film materials samples, which are based on allantoin-modified sodium salt of carboxymethylcellulose (**2**, **3**), exhibit a diminished ability for macrophages from the RAW 264.7 line to adhere, and their adhesion value is comparable to that of sample (**1**).

The samples of composite film materials, derived from the glycoluril-modified sodium salt of carboxymethylcellulose (**4**, **5**), exhibit notably reduced adhesion of RAW 264.7 line macrophages to the surface compared to both sample (**1**) and the examined composite film materials based on allantoin-modified sodium salt of carboxymethylcellulose (**2**, **3**).

### 2.5. Evaluation of Antibacterial Activity

Following the examination of antibacterial activity, it was observed that the samples from all groups, including the control sample of film (**1**), did not exhibit any noteworthy activity against the test strains.

### 2.6. Evaluation of Antioxidant Activity

In the experiments measuring antioxidant activity, it was determined that the commercial sample (**1**) decreased the concentration of ABTS cation radical in the model system, equivalent to 2.19 ± 0.32 μmol of Trolox (Table 5). The investigated samples of composite film materials, based on the sodium salt of carboxymethylcellulose modified with allantoin (**2**, **3**), exhibited more pronounced antioxidant effects (5.49 ± 0.47 and 9.99 ± 0.51 μmol of Trolox equivalents/g film, respectively). The samples of composite film materials, derived from the glycoluril-modified sodium salt of carboxymethylcellulose (**4**, **5**), demonstrated the highest antioxidant activity (10.02 ± 0.86 and 21.27 ± 0.91 μmol of Trolox equivalents/g film, respectively)

Hence, all experimental film material samples based on Na-CMC modified with glycoluril and allantoin (**2**–**5**) exhibit superior antioxidant activity compared to the membrane sample (**1**), demonstrating the capability to diminish the level of free radicals in model systems using DPPH and ABTS radicals. The samples of composite film materials based on Na-CMC modified with glycoluril (**4**, **5**) display the most pronounced antioxidant effect.

### 2.7. Investigation of the Mechanical Properties of Film Materials

In experiments measuring the mechanical strength of samples, it was established that samples based on the sodium salt of carboxymethylcellulose modified with allantoin (**2**, **3**) and glycoluril (**4**, **5**) have a tensile strength higher than the reference sample “Seprafilm” (Table 6).

### 2.8. Investigation of Water Content in the Obtained Film Materials

The water content of the samples of film materials modified with allantoin (**3**) and glycoluril (**5**) was determined to be residual moisture. Samples (**3**) and (**5**) did not demonstrate high humidity (0.27% and 0.23%, respectively). 

## 3. Discussion

In this study, composite film materials based on allantoin and glycoluril were synthesized for the first time, and consequently, their beneficial biological properties have not yet been established. To gauge the potential of these biomaterials in medical applications, it is imperative to initially assess their biocompatibility. The convergence of favorable biocompatible traits in the examined material paves the way for the potential development of targeted medical products. The outcomes of the biocompatibility assessment for the developed biomaterials are presented and discussed in the following sections. 

### 3.1. Evaluation of Hemocompatibility of Composite Film Materials

As indicated in Table 1, the experimental film samples do not exhibit a lytic effect on erythrocytes, and their hemolytic activity is comparable to that of the Seprafilm™ reference sample (**1**).

Hemolysis refers to the temporary or permanent damage to red blood cells (erythrocytes), resulting in the diffusion leakage of hemoglobin (Hb) from the cells. Various factors, including mechanical, thermal, chemical, and biological influences, can contribute to red blood cell damage when they come into contact with devices handling blood. Mechanical hemolysis, for instance, can occur during the flow transport of red blood cells in medical devices such as syringe pumps [26], artificial hearts [27], and heart valves [28]. Prolonged contact and collision between blood cells and the surfaces of foreign devices, particularly barrier films, can lead to erythrocyte damage. When erythrocytes are damaged, hemoglobin is released into the plasma, which, in severe cases, can result in renal failure, anemia, arrhythmias, and even death. According to the literature, hemolysis on inert biomaterial surfaces is directly linked to the adsorption of plasma proteins, particularly fibrinogen, onto the material’s blood-contacting surface. The extent of plasma protein adsorption correlates with the degree of hemolysis [29]. Considering these factors, this study focused on investigating the adsorption of blood plasma proteins on the surface of the examined composite film materials.

### 3.2. Adsorption of Blood Plasma Proteins on the Surface of the Investigated Composite Film Materials

As indicated by the data in Table 2, the protein adsorption in the reference sample (**1**) was measured at 2.96 ± 0.13%. The protein adsorption in the investigated composite film materials, which are based on glycoluril- and allantoin-modified sodium salt of carboxymethylcellulose (**2**–**5**), ranged from 3.06 ± 0.17% to 3.28 ± 0.17%, with *p*-values of 0.917, 0.117, 0.347, and 0.117, respectively. These values were not significantly different from the protein adsorption capacity of the reference sample (**1**).

The challenge of undesired blood clotting upon contact with implanted materials and devices remains unresolved. This issue arises because healthy vascular endothelium possesses mechanisms that resist thrombosis, while foreign materials lack such protective mechanisms. Instead, biomaterials stimulate blood clotting through the activation of interconnected processes, including protein adsorption, thrombocyte and leukocyte adhesion, thrombin production, and complement system activation [29]. Simultaneously, heightened adsorption of plasma proteins results in the accumulation of fibrinogen on the surface of the implanted material. Fibrinogen serves as a protein precursor of fibrin, forming the foundation of the clot during hemostasis. Disruption of the balance between fibrin deposition and degradation is a critical factor in adhesion formation [30]. In this context, the quest for biocompatible materials that do not undergo surface adsorption becomes particularly crucial. 

### 3.3. Cytotoxicity

An additional crucial aspect of biocompatibility involves the in vitro cytotoxicity of materials. The cytotoxicity of the samples was assessed using the 3T3 cell line, which is obtained from mouse fibroblasts. Fibroblasts play a role in secreting collagen proteins, contributing to the maintenance of the structural framework in numerous tissues. Dermal fibroblasts, in particular, are responsible for producing connective tissue and extracellular matrix components that influence epidermal regeneration and facilitate wound healing [31]. 

Following the experiments, it was observed that the incubation of the normal fibroblast cell line 3T3-L1 with various extract concentrations of the investigated samples of composite film materials based on allantoin-modified sodium salt of carboxymethylcellulose (**2**, **3**) for 24 h, as well as the comparison sample (**1**), did not result in a significant decrease in cell viability in the neutral red test (Figure 2). 

Similarly, incubation of the normal fibroblast cell line 3T3-L1 with different extract concentrations of the examined samples of composite film materials based on glycoluril-modified sodium salt of carboxymethylcellulose (**4**, **5**) for 24 h showed no impact on cell viability (Figure 3).

Therefore, the extracts from the studied films, as well as the extracts from the comparison film, do not exhibit a notable cytotoxic effect against the culture of 3T3-L1 fibroblast cells. 

A comparative analysis of the cytotoxicity of the investigated samples of composite film materials (**2**–**5**) and the comparison sample, Seprafilm™ membrane (Genzyme Corporation, Cambridge, MA, USA), is presented in Table 3 and Figure 4. The results indicate that the cytotoxicity of the studied samples of composite film materials, based on the sodium salt of carboxymethylcellulose modified with glycoluril and allantoin (**2**–**5**), does not differ from the cytotoxicity of sample (**1**).

### 3.4. Adhesion of Macrophages to the Surface of the Tested Samples 

Following the investigation of cytotoxicity, the adhesion of macrophages to the surface of the examined samples was scrutinized. Experimental results revealed that the number of adhered macrophages of the RAW 264.7 line on the surface of tablet wells lacking the investigated composite film materials within the microscope’s field of view was 355 ± 36 (Table 4). Upon placing RAW 264.7 cells in the wells of the tablet on the film surface of the comparison sample “Seprafilm”, the number of adhered cells significantly decreased to 128 ± 16 in the microscope’s field of view. The extent of macrophage adhesion on the surface of the samples of composite film materials based on allantoin-modified sodium salt of carboxymethylcellulose (**2**, **3**) did not differ from sample (**1**), with the number of adhered cells in the microscope’s field being 215 ± 31 (*p* = 0.058) and 195 ± 29 (*p* = 0.064), respectively. The ability to adhere macrophages in the samples of composite film materials based on glycoluril-modified sodium salt of carboxymethylcellulose (**4**, **5**) was significantly lower than in sample (**1**) and the studied samples (**2**, **3**), with the number of adhered cells in the microscope’s field being 61 ± 8 and 18 ± 5, respectively.

Hence, the samples of the examined composite film materials based on allantoin-modified sodium salt of carboxymethylcellulose (**2**, **3**) exhibit a limited ability to adhere RAW 264.7 macrophages, and their adhesion capability is not inferior to that of sample (**1**). In the case of samples of composite film materials based on glycoluril-modified sodium salt of carboxymethylcellulose (**4**, **5**), the adhesion of RAW 264.7 macrophages to the surface is significantly lower compared to sample (**1**) and the investigated composite film materials based on allantoin-modified sodium salt of carboxymethylcellulose (**2**, **3**).

In a well-functioning organism, the innate immune system serves as the initial defense against both external and internal stress signals. The mononuclear phagocyte system, comprising circulating monocytes, resident macrophages, and dendritic cells, assumes a crucial role in inflammation, defense against pathogens, and the direct elimination of foreign agents. 

Macrophages are distributed across various tissues in the human body and play a pivotal role in promptly responding to the invasion of foreign pathogens. They constitute a vital component of the innate immune system, actively participating in the initiation and execution of responses within the acquired immune system. Dysfunction of macrophage functions can result in the development of chronic inflammation and autoimmune diseases and contribute to the progression of cancer. Tissue-resident macrophages demonstrate the ability to swiftly adapt to environmental triggers by modulating gene expression [32]. This rapid response, typically observed during inflammation or tissue damage, is referred to as macrophage activation, involving an increased production of cytokines, chemokines, and other inflammatory mediators. This, in turn, facilitates the recruitment of additional macrophages [32].

Macrophages, acknowledged as crucial effector cells within the innate immune system, serve not only as the primary defense against microorganisms but also play a key role in triggering and regulating adaptive immune responses [33]. Consequently, macrophages warrant significant consideration when examining the pathogenetic aspects of diseases characterized by an inflammatory component [32]. 

One crucial aspect of medical materials is their cytotoxicity and impact on the immune system. According to the literature findings, immune system cells play a pivotal role in adhesion formation. Specifically, macrophages release various factors influencing the healing processes of the peritoneum post-surgery, thereby modulating the inflammatory response across a significant area of the wound surface [30].

It can be inferred that increased macrophage adhesion to implanted material is linked to the activation of the inflammation process resulting from tissue trauma. Consequently, the higher the macrophage adhesion to the biomaterial surface, the greater the likelihood of inflammatory and, subsequently, adhesion process development. It can also be hypothesized that the level of macrophage adhesion to the biomaterial surface serves as an indicator of the material’s toxicity to immune system cells. Higher macrophage adhesion implies higher toxicity and, consequently, an increased probability of adhesion formation. The subsequent stage of the research will involve studying the impact of the developed composite biomaterials on macrophage activation and assessing their immunomodulatory properties.

### 3.5. Antibacterial Activity

As a result of experiments to study the antibacterial activity of film samples, it was established that there were no zones of inhibition of the growth of test strains around the samples (*d* = 0). It follows that samples from all groups, including the control film sample (**1**), did not show any noticeable activity against the test strains. The absence of antibacterial activity suggests that the films are not conducive to the growth of microorganisms, indicating that bacteria do not utilize them as a nutrient substrate.

### 3.6. Antioxidant Activity

In the examination of antioxidant activity, it was observed that the commercial “Seprafilm” membrane sample reduced the concentration of the ABTS cation radical in the model system, equivalent to 2.19 ± 0.32 μmol of Trolox (Table 5). The investigated composite film materials based on allantoin-modified sodium salt of carboxymethylcellulose (**2**, **3**) displayed a more notable antioxidant effect (5.49 ± 0.47 and 9.99 ± 0.51 μmol Trolox equivalents/g film, respectively). Furthermore, samples of composite film materials based on glycoluril-modified sodium salt of carboxymethylcellulose (**4**, **5**) exhibited the highest antioxidant activity (10.02 ± 0.86 and 21.27 ± 0.91 μmol of Trolox equivalents/g film, respectively).

Consequently, all experimental samples of composite film materials based on Na-CMC modified with glycoluril and allantoin (**2**–**5**) showcase superior antioxidant activity compared to the “Seprafilm” membrane sample (Genzym Corporation, Cambridge, MA, USA). They demonstrate the capacity to reduce the level of free radicals in model systems using DFPH and ABTS radicals. The most significant antioxidant activity is observed in samples of composite film materials based on Na-CMC modified with glycoluril (**4**, **5**).

### 3.7. Mechanical Properties of Film Materials

In the examination of mechanical strength, it was noted that the studied composite film materials based on allantoin- and glycoluril-modified sodium salts of carboxymethylcellulose (**2**–**5**) showed higher tensile strength than the commercial sample (**1**) (Table 6). Furthermore, samples of composite film materials based on Na-CMC modified with glycoluril and allantoin (**2**, **4**) exhibited the highest tensile strength (47.51 and 46.40 MPa, respectively).

### 3.8. Water Content in the Obtained Film Materials

As a result of experiments to study the water content in the obtained film materials, it was established that composite film materials based on Na-CMC modified with allantoin (**3**) and glycoluril (**5**) did not consist of high water content. However, composite film materials based on allantoin-modified sodium salt of carboxymethylcellulose (**3**) contain more water than composite film materials based on Na-CMC modified with glycoluril (**5**) (0.27% and 0.23%, respectively). 

## 4. Materials and Methods

### 4.1. Production of Composite Film Materials Based on Carbamide-Containing Heterocycles

#### 4.1.1. Production of Allantoin-Based Composite Film Materials (**2**, **3**)

##### Preparation of Allantoin (2,5-Dioxo-4-imidazolidinyl) Urea

A total of 1.80 g of 50% aqueous urea solution, 0.25 mL (0.41 g) of 50% phosphoric acid solution, and 0.02 g of sulfamic acid are placed in a flask equipped with a stirrer and a reflux condenser. Then, 1.13 mL (1.5 g) of 50% glyoxalic acid solution is added dropwise while stirring at 15 °C. The mixture is stirred for 2 h at 95 °C; in this case, the formation of white precipitate of allantoin is observed. After cooling, the mixture is filtered, washed with warm water, and dried. The output is (64%) [24,34]. Melting point is 225 °C (with decomposition). ^1^H NMR spectrum (δ, ppm, DMSO-d6): 8.05 (^1^H, s), 6.94 (^1^H, d), 5.83 (^2^H, s), 5.24 (^1^H, d). ^13^C NMR spectrum (δ, ppm, DMSO-d6): 173.79 (C=O), 157.70 (C=O), 157.06 (C=O), 62.61 (C-tert.). IR spectrum (cm^−1^): 3436 (NH_2_), 3068 (NH_2_), 3192 (NH), 2947 (CH), 1780 (C=O), 1719 (C=O), 1667 (C=O), 1602 (NH_2_), 1430 (NH).

##### Production of a Film Material Containing Allantoin (**2**)

Adding an aqueous solution of allantoin with a mass fraction of 0.01% to 0.2 g of the film-forming agent Na-CMC M.W. 250,000 (DS = 0.9, Acros Organics, Geel, Belgium) or Na-CMC M.W. 250,000 (DS = 1.2) up to a total mass of 10 g. The resulting solutions are dispensed onto the substrate (10 mL) and air-dried for 24 h. 

##### Production of a Film Material Containing Allantoin (**3**)

Adding an aqueous solution of allantoin with a mass fraction of 0.05% to 0.2 g of the film-forming agent Na-CMC M.W. 250,000 (DS = 0.9, Acros Organics, Belgium) or Na-CMC M.W. 250,000 (DS = 1.2) up to a total mass of 10 g. The resulting solutions are dispensed onto the substrate (10 mL) and air-dried for 24 h. 

#### 4.1.2. Production of Composite Film Materials Based on glycoluril (**4**, **5**)

##### Production of glycoluril (2,4,6,8-tetraazabicyclooctane[3.3.0]octane-3,7-dione)

To an aqueous solution of 6.0 g (0.1 mol) of urea and 7.3 g of 40% aqueous solution of glyoxal (0.05 mol), 20.6 g (0.1 mol) of OEDP is added while stirring. The reaction mixture is heated to 80 °C and incubated for one hour. After 10 min, the solution starts to become turbid, and the product precipitates. After 40–50 min, the reaction mixture is cooled, and the precipitate is filtered and washed with water [25]. The substance is a white powder, and the yield is 99%. Temperature of decomposition is more than 360 °C. ^1^H NMR spectrum (δ, ppm, DMSO-d6): 7.17 (s. ^4^H, NH), 5.24 (s. ^2^H, CH). ^13^C NMR spectrum (δ, ppm, DMSO-d6): 161.72 (C=O), 65.05 (CH).

##### Production of a Film Material Containing Glycoluril (**4**)

Adding an aqueous solution of glycoluril with a mass fraction of 0.01% to 0.2 g of the film-forming agent Na-CMC M.W. 250,000 (DS = 0.9, Acros Organics, Belgium) or Na-CMC M.W. 250,000 (DS = 1.2) up to a total mass of 10 g. The resulting solutions are dispensed onto the substrate (10 mL) and air-dried for 24 h.

##### Production of a Film Material Containing Glycoluril (**5**)

Adding an aqueous solution of glycolurol with a mass fraction of 0.05% to 0.2 g of the film-forming agent Na-CMC M.W. 250,000 (DS = 0.9, Acros Organics, Belgium) or Na-CMC M.W. 250,000 (DS = 1.2) until the total mass of the solution reaches 10 g. The resulting solutions are dispensed onto the substrate using a dispenser (10 mL) and air-dried for 24 h.

### 4.2. Evaluation of Hemocompatibility of Composite Film Materials

Hemocompatibility of the samples was evaluated by comparing the optical density of the extract suspension with blood to the optical density of blood under conditions of 100% hemolysis [35].

To assess the hemocompatibility of the samples, a set of extracts was created from the examined composite film materials derived from Na-CMC modified with glycoluril and allantoin. Sterilized penicillin vials were filled with 4 mL of sterile physiological solution each. Samples of the investigated composite film materials, along with the Seprafilm™ comparison sample, were positioned in a [1.5 × 1.5] cm area and subjected to incubation in the thermostat at 37 °C for 24 h.

Blood was drawn from five healthy donors (5 mL) into vacuum vials containing an anticoagulant (citrate) and was then centrifuged at 900× *g* for 15 min at 4 °C following a 2 h incubation at room temperature. The resulting supernatant was separated, and 8 mL of 0.9% sodium chloride solution was added to the precipitate. After gently shaking and centrifuging at 900× *g* for 10 min at 4 °C, the supernatant was once again separated, and red blood cell washing was repeated two additional times. For the creation of a 10% red cell suspension, 1 mL of red cell mass was combined with 9 mL of 0.9% sodium chloride solution. This resulting erythrocyte suspension was stored at 4 °C for no more than 24 h.

Control samples comprised 0.2 mL of erythrocyte suspension mixed with 2 mL of 0.9% sodium chloride solution. For samples with 100% hemolysis, 0.2 mL of erythrocyte suspension was combined with 2 mL of distilled water, resulting in complete destruction of erythrocytes, corresponding to 100% hemolysis. Experimental samples consisted of 0.2 mL of erythrocyte suspension and 2 mL of previously prepared extracts from the investigated samples. These samples were then placed in a thermostat at 37 °C for 1 h. Subsequently, tubes containing experimental samples, control samples, and samples with 100% hemolysis underwent centrifugation using a Hermle Z 383 K centrifuge for 20 min at a speed of 6000 rpm. The resulting supernatant was separated for optical density measurement using an SF-2000 spectrophotometer (Saint Petersburg, Russia).

The optical density of control samples, consisting of a 10% erythrocyte suspension with 0.9% sodium chloride solution, should not surpass 0.03 optical units. Meanwhile, the optical density of samples indicating 100% hemolysis should range from no less than 0.8 to no more than 1.0 optical units [35].

### 4.3. Adhesion Evaluation of Blood Serum Proteins to the Surface of Composite Film Materials

To assess the adhesion of serum proteins, whole anticoagulated blood from a healthy donor was employed. Following a 2 h incubation at room temperature, the blood was centrifuged at 600× *g* for 15 min at 4 °C. The resulting serum was diluted tenfold with 0.9% NaCl, and the protein concentration in each sample was spectrophotometrically measured using the Bradford method with Coomassie G-250 dye [36]. A calibration curve was established using bovine serum albumin (BSA) as a standard.

The investigated composite film materials were positioned in 24-well culture dishes with a surface area of 1.9 cm². Subsequently, 300 µL of 0.9% NaCl was added to the wells and allowed to swell the composite films for 3 min, after which the residual 0.9% NaCl was removed. Following this, tenfold diluted serum was added to each well, and the composite film materials were incubated in a thermostat for 2 h at 37 °C. After incubation, serum was withdrawn from the wells, and the concentration of unadsorbed proteins was measured through a spectrophotometric method.

The percentage of protein adsorbed by the films was calculated by the difference between the total protein content of serum and the amount of unadsorbed proteins after incubation with the films.

### 4.4. Evaluation of Cytotoxicity of Composite Film Materials

The cytotoxicity towards fibroblasts was evaluated using the extract method [37]. To assess the cytotoxic activity of the tested samples of composite film materials based on the sodium salt of carboxymethylcellulose modified with glycoluril and allantoin, as well as the comparison sample Seprafilm™, a colorimetric test utilizing the intracellular uptake of the vital dye neutral red was selected [38,39].

Before the experiment, cells of the 3T3-L1 cell line (obtained from FSIS SRC VB “Vector” Rospotrebnadzor) were cultured in complete nutrient medium (DMEM/F-12, 292 mg/L L-glutamine, 50 mg/L gentamicin, 10% FBS) for at least 3 passages.

Briefly, after passaging of cells in 96-well plates at 10,000 per well, the plates were kept in a CO_2_ incubator (Sanyo, Tokyo, Japan) for 24 h for cell adhesion and to achieve 80–90% confluence. During this period, extracts of the examined samples were generated as follows: 1.5 × 1.5 cm composite films were positioned under aseptic conditions in a pre-sterilized penicillin vial containing 4 mL of complete nutrient medium (CNM) and incubated for 24 h at 37 °C to produce a 100% extract. The 50% and 25% extracts were derived through successive 2-fold dilutions of the 100% CNM extract.

On the following day, the medium in the cell-containing wells was substituted with extracts from the examined composite film samples (100 μL). Control cells received an equivalent volume of CNM.

After substituting the CNM with extracts in the wells, the plates containing cells were placed in a CO_2_ incubator for 24 h. Following incubation, extracts were aspirated from the wells, cells were rinsed once with 200 μL of 1X PBS, and 100 μL of CNM with neutral red (40 μg/mL) was dispensed into each well. The plates were incubated in a thermostat for 2 h at 37 °C. Afterward, the incubation medium containing the dye was carefully aspirated, cells were washed once with 200 µL of 1X PBS, and 150 µL of a mixture of 96% ethanol: deionized water: glacial acetic acid (50:49:1) was added to each well to extract the bound dye. The plate was positioned in a Tecan Sunrise microplate spectrophotometer, and the optical density in each well was measured at 540 nm with a reference wavelength of 650 nm.

Cell viability post-incubation with extracts of composite film samples was computed relative to cell viability in the control and expressed as a percentage of viable cells.

Statistical analysis of in vitro experiment data was conducted using GraphPad Prism7 (GraphPad Software, Inc., La Jolla, CA, USA) and SPSS 17.0 (IBM, Armonk, NY, USA) software packages. Descriptive statistics, including the mean and standard deviation, were computed for all data. The normality of distribution was assessed using the Shapiro–Wilks test.

### 4.5. Evaluation of Macrophage Adhesion to the Surface of Composite Film Materials

The test films or the Seprafilm comparison sample were positioned in 24-well culture plates, fully covering the bottom surface of each well. DMEM/F-12 medium supplemented with 10% fetal bovine serum and 10 μg/mL gentamicin was introduced into the wells. After allowing the plates to stand for 3 min to facilitate swelling of the composite films, any remaining medium was removed. Subsequently, a suspension of RAW 264.7 macrophages (ATCC, Manassas, VA, USA) in DMEM/F-12 complete nutrient medium was added to the wells, achieving a concentration of 50 × 103 cells per 1 cm^2^ of the surface of the investigated composite film materials.

The plates were subjected to a 24 h incubation at 37 °C in a 5% CO_2_ atmosphere. Following this, non-adherent cells were removed by resuspending the cell suspension. Cells attached to the surface of the composite film materials were subsequently fixed with 2.5% glutaraldehyde for 30 min and stained with fluorescent dyes, specifically Phalloidin-Atto-488 for cytoskeleton staining and DAPI for nucleus staining.

The count of cells adhered to the surface of the composite film materials within the microscope’s field of view was conducted utilizing a Leica DMi8 fluorescence microscope [40]. Three repetitions of experiments were carried out for each composite film sample and the comparison sample “Seprafilm”, with the number of adhered cells in each experiment assessed across three fields of view.

### 4.6. Evaluation of Antibacterial Activity of Composite Film Materials

The experiment focused on examining the antibacterial activity of polymer films modified with heterocyclic nitrogen-containing compounds, namely allantoin and glycoluril. These samples served as prototypes for films designed to prevent adhesion. Four groups of polymer film samples (**2**–**5**) were investigated during the experiment. For comparison, a film with a similar purpose from industrial production, “Seprafilm” from the USA (**1**), was used as a positive control.

The evaluation of the antibacterial activity of the samples was conducted through a modified version of the standard disk-diffusion method, where the sample was applied to the surface of a dense agarized medium. The modifying agents from the samples diffused into the medium, leading to the formation of zones indicating the suppression of bacterial growth.

To investigate the impact of the samples on both Gram-positive and Gram-negative microflora, *Staphylococcus aureus* and *Escherichia coli* ATCC 25922 (American Type Culture Collection) were employed as the test subjects.

The liquid medium utilized for the cultivation of *Escherichia coli*, a standard for this test object, was LB medium comprising the following concentrations in g/L: peptone—10; yeast extract—5; and sodium chloride—10. The incubation process took place in a thermostat within 250 mL Erlenmeyer flasks containing 100 mL of LB medium at a temperature of 37 °C.

The liquid medium used for the cultivation of *Staphylococcus aureus*, a standard for this test object, was fish meal hydrolysate bouillon consisting of the following concentrations in g/L: fish meal hydrolysate—8; meat peptone—8; and sodium chloride—4. Incubation occurred in a thermostat within 250 mL Erlenmeyer flasks containing 100 mL of the medium at a temperature of 37 °C.

The solid medium used for sowing *Staphylococcus aureus*, modified by the author, was fish egg meal hydrolysate agar. This medium contained the following concentrations in g/L: fish meal hydrolysate—8; meat peptone—8; sodium chloride—4; egg yolk solution—200 mL; and bacteriological agar—1.5–2% of the volume. Incubation took place in a thermostat at 37 °C.

The solid medium used for sowing Escherichia coli, following the standard for this test object, was LB medium. This medium contained the following concentrations in g/L: peptone—10; yeast extract—5; sodium chloride—10; and bacteriological agar—1.5–2% of the volume. Incubation occurred in the thermostat at 37 °C.

The test strain was uniformly spread on each Petri dish containing 15 mL of a suitable nutrient-rich medium using the lawn method from a pure culture. Subsequently, a sample was positioned at the center of each dish. Following incubation, the diameter of the zone where bacterial growth was inhibited was measured with a precision of 0.1 mm. A larger zone indicated greater antibacterial activity of the sample, and the zone of bacterial growth inhibition was identified as the region where colony growth was completely suppressed.

### 4.7. Evaluation of Antioxidant Activity of Composite Film Materials

The determination of the films’ antioxidant activity involved a spectrophotometric assessment based on the reduction in the concentration of the ABTS cation radical in the reaction medium. The reaction mechanism of the model ABTS cation radical encompasses both hydrogen atom recoil and electron transfer [41].

The ABTS radical was generated in the solution through the action of potassium peroxodisulfate. To achieve this, a stock solution of ABTS was prepared by dissolving a (11 ± 0.1) mg suspension of ABTS in 900 μL of distilled water. Simultaneously, a (20 ± 0.1) mg suspension of potassium peroxodisulfate was dissolved in 1 mL of distilled water. Subsequently, 100 μL of the potassium peroxodisulfate stock solution was added to 900 μL of the ABTS solution.

To create the working solution, the ABTS cation radical stock solution was diluted in 0.1 M phosphate buffer (pH 7.4) until reaching an optical density of 0.70 ± 0.02 at 734 nm, with an optical path length of 1 cm. It is important to note that the ABTS cation radical working solution is not stable and was freshly prepared before each use.

A 400 μM solution of Trolox, a water-soluble analog of tocopherol, was utilized to create a calibration curve through a series of dilutions. It is worth noting that Trolox solutions are not stable and were freshly prepared just before usage.

During the construction of the calibration curve, 200 µL of each Trolox solution concentration was introduced into 1.8 mL of the ABTS radical working solution. After a 40 min incubation, the optical density of the solutions was measured spectrophotometrically at 734 nm. A calibration curve was then generated using the mean values obtained for various Trolox standard concentrations.

To assess antioxidant activity, the examined composite film materials measuring 1.5 × 1.5 cm were submerged in a 2 mL working solution of ABTS cation radical. After a 40 min incubation, the optical density of the solutions was recorded. Antioxidant activity was quantified in units of equivalent concentration of the water-soluble analog of tocopherol, Trolox, utilizing a calibration curve.

### 4.8. Investigation of the Mechanical Properties of Film Materials

In order to study the mechanical properties of the composite biomaterial samples, an Instron 3347 tensile machine has been used.

After drying the composition, the obtained films were cut into rectangles of 50 mm long and 30 mm wide. The geometric dimensions of the samples were measured to an accuracy of 0.1 mm. Thickness of each sample was measured on three points using a digital micrometer, and its accuracy was 0.01 mm.

### 4.9. Investigation of Water Content in the Obtained Film Materials

For the obtained film material samples, the water content was determined according to K. Fisher. The experiment was carried out in accordance with the methodology [42].

## 5. Conclusions

This study examined the biocompatibility of the developed composite materials, considering their hemolytic impact, cytotoxicity, and antibacterial and antioxidant activities, as well as the adhesion of blood serum proteins and macrophages to the surface. Comparative analysis revealed that the proposed film materials incorporating allantoin and glycoluril exhibit biocompatible properties comparable to, if not superior to, the commercial reference medical product “Seprafilm”. Notably, a significant experimental finding was the low adhesion of macrophages to the surface of composite film materials based on glycoluril, coupled with their elevated antioxidant activity, surpassing the values observed for the commercial sample “Seprafilm”.

The promising biocompatibility findings of the newly developed composite film materials based on glycoluril suggest a favorable avenue for their practical application in abdominal surgery.

## Figures and Tables

**Table 1:** The effect of extracts of film samples on erythrocyte hemolysis.

Samples	№	Hemolysis, %				
		Erythrocyte Suspension №1	Erythrocyte Suspension №2	Erythrocyte Suspension №3	Erythrocyte Suspension №4	Erythrocyte Suspension №5
**1**	1.	0.59	0.70	0.29	0.57	0.47
	2.	0.38	0.20	0.87	0.43	0.49
	3.	0.39	0.32	0.23	0.56	0.64
	X ± m	0.45 ± 0.07	0.41 ± 0.15	0.46 ± 0.20	0.52 ± 0.04	0.53 ± 0.06
**2**	1.	0.40	0.59	0.40	0.29	0.44
	2.	0.47	0.13	0.99	0.81	0.64
	3.	0.77	0.43	0.38	0.80	0.31
	X ± m	0.55 ± 0.11	0.38 ± 0.13	0.59 ± 0.20	0.63 ± 0.17	0.46 ± 0.09
**3**	1.	0.66	1.04	0.70	0.41	0.66
	2.	0.25	0.50	0.34	0.69	0.33
	3.	0.67	0.45	0.35	0.46	0.57
	X ± m	0.52 ± 0.14	0.66 ± 0.19	0.46 ± 0.12	0.52 ± 0.09	0.52 ± 0.09
**4**	1.	0.38	0.47	0.28	0.82	0.53
	2.	0.40	1.22	1.17	0.46	0.37
	3.	0.61	0.57	0.55	0.38	0.59
	X ± m	0.46 ± 0.07	0.75 ± 0.23	0.67 ± 0.26	0.55 ± 0.14	0.49 ± 0.07
**5**	1.	0.47	0.49	0.45	0.36	0.40
	2.	0.63	0.74	0.67	0.60	0.66
	3.	0.36	1.14	0.40	0.76	1.68
	X ± m	0.49 ± 0.08	0.79 ± 0.19	0.51 ± 0.08	0.57 ± 0.12	0.91 ± 0.39

**Table 2:** The adsorption of proteins on the surface of the investigated films.

Samples	№	Adsorption of Proteins, %				
		Serum №1	Serum №2	Serum №3	Serum №4	Serum №5
**1**	1.	2.36	2.94	1.81	3.80	1.54
	2.	4.41	2.82	2.71	3.51	3.68
	3.	2.88	3.42	3.08	2.17	3.20
	X ± m	2.96 ± 0.13				
**2**	1.	1.48	3.04	1.89	4.82	4.16
	2.	2.76	2.69	3.86	3.05	1.96
	3.	4.02	3.55	2.41	3.16	3.01
	X ± m	3.06 ± 0.17 *p*_2–1_ = 0.917				
**3**	1.	2.15	2.47	4.08	5.60	3.71
	2.	3.83	3.42	1.74	3.55	4.38
	3.	3.37	4.15	3.08	1.32	2.47
	X ± m	3.29 ± 0.11 *p*_3–1_ = 0.117				
**4**	1.	4.81	3.10	2.93	4.33	3.14
	2.	3.01	2.63	2.22	2.73	2.21
	3.	0.83	4.24	5.35	2.20	3.74
	X ± m	3.16 ± 0.11 *p*_4–1_ = 0.347				
**5**	1.	2.73	3.07	3.00	2.45	3.74
	2.	3.68	4.02	6.05	2.98	1.54
	3.	3.16	0.94	1.18	5.57	5.08
	X ± m	3.28 ± 0.17 *p*_5–1_ = 0.117				

**Table 3:** Cytotoxicity rates of various extract concentrations determined by neutral red assay.

Film Sample	Extract, %	Cell Viability, %, M ± SD	Reliability of Differences
**1**	25%	98.1 ± 5.6	
	50%	96.9 ± 7.8	
	100%	93.0 ± 8.6	
**2**	25%	99.4 ± 6.2	*p* = 0.344
	50%	97.9 ± 6.9	*p* = 0.612
	100%	96.5 ± 6.9	*p* = 0.356
**3**	25%	96.9 ± 3.3	*p* = 0.450
	50%	95.4 ± 4.3	*p* = 0.220
	100%	94.7 ± 5.4	*p* = 0.875
**4**	25%	97.9 ± 4.9	*p* = 0.831
	50%	95.2 ± 5.4	*p* = 0.177
	100%	94.9 ± 8.9	*p* = 0.982
**5**	25%	97.2 ± 4.9	*p* = 0.685
	50%	95.6 ± 4.2	*p* = 0.558
	100%	96.0 ± 5.9	*p* = 0.620

Note: *p* reflects the reliability of differences from the comparison film–commercial sample of Seprafilm™ membrane (Genzym Corporation, USA).

**Table 4:** Adhesion of macrophages of the RAW 264.7 cell line to the surface of the samples of the tested films.

Film Samples	Phalloidin	DAPI	Merge	RAW 264.7 Value
**Control (n = 9)**	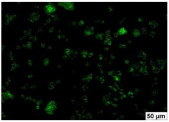	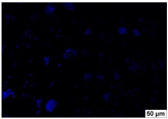	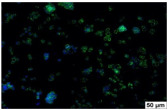	355 ± 36
**1** **(n = 9)**	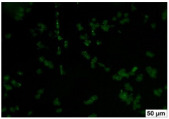	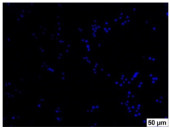	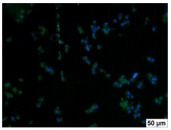	128 ± 16 *p*_2–1_ < 0.001
**2** **(n = 9)**	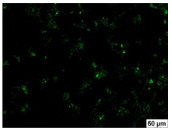	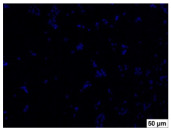	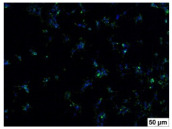	215 ± 31 *p*_3–1_ < 0.03 *p*_3–2_ = 0.058
**3** **(n = 9)**	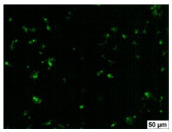	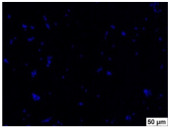	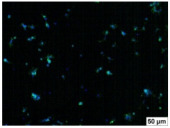	195 ± 29 *p*_4–1_ < 0.002 *p*_4–2_ = 0.064 *p*_4–3_ = 0.757
**4** **(n = 9)**	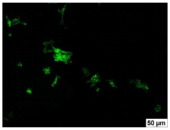	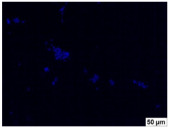	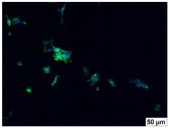	61 ± 8 *p*_5–1_ < 0.001 *p*_5–2_ < 0.005 *p*_5–3_ < 0.001
**5** **(n = 9)**	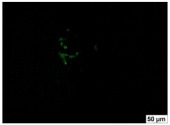	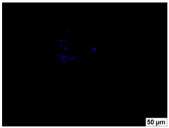	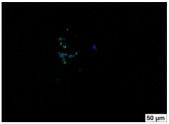	18 ± 5 *p*_6–1_ < 0.001 *p*_6–2_ < 0.001 *p*_6–4_ < 0.001 *p*_6–5_ < 0.002

**Table 5:** Antioxidant activity of film samples expressed in Trolox equivalents.

Film Samples	Antioxidant Activity, µmol Trolox/g Film
**1** (n = 6)	2.19 ± 0.32
**2** (n = 6)	5.49 ± 0.47 (*p*_2–1_ < 0.005)
**3** (n = 6)	9.99 ± 0.51 (*p*_3–1_ < 0.001)
**4** (n = 6)	10.02 ± 0.86 (*p*_4–1_ < 0.001)
**5** (n = 6)	21.27 ± 0.91 (*p*_5–1_ < 0.001)

**Table 6:** Mechanical properties of film samples.

Film Samples	Tensile Strength of Films, MPa
**1**	32.02
**2**	47.51
**3**	43.36
**4**	46.40
**5**	40.58

## Data Availability

The data presented in this study are available in this article.
